# SlERF.D2-mediated antagonism between ethylene and ABA signaling pathways modulates osmotic stress adaptation in tomato

**DOI:** 10.1093/hr/uhaf267

**Published:** 2025-10-13

**Authors:** Ning Li, Fan Lu, Benke Kuai

**Affiliations:** State Key Laboratory of Genetic Engineering and Fudan Center for Genetic Diversity and Designing Agriculture, School of Life Sciences, Fudan University, Shanghai 200438, China

## Abstract

Ethylene response factors (ERFs) are pivotal regulators in mediating plant stress adaptation; however, the roles of osmotic stress-responsive ERFs in tomato remain poorly characterized. Here, we comprehensively investigate the function of SlERF.D2, a member of the ERF transcription factor family, in modulating osmotic stress adaptation. Expression profiling indicated that *SlERF.D2* responded to diverse abiotic stimuli, such as drought and salt, as well as ethylene and abscisic acid (ABA). Combined physiological and metabolomic analyses of *SlERF.D2* overexpression and knockout lines revealed a negative regulatory role of SlERF.D2 in tomato's osmotic stress adaptation. Biochemical and molecular assays further revealed that SlERF.D2 directly targets the promoter of *SlPP2C1*, an ABA signaling suppressor, to activate its expression, thereby impairing ABA-dependent stomatal closure and accelerating water loss. Notably, ethylene-induced *SlERF.D2* expression required the direct binding of SlEIL1/2/3/4 to the *SlERF.D2* promoter. Furthermore, ethylene activated *SlPP2C1* transcription in an SlERF.D2-dependent manner through direct transcriptional regulation by SlERF.D2. Thus, the ethylene-SlEIL1/2/3/4-SlERF.D2-SlPP2C1 transcriptional cascade module is involved in the antagonism of ABA-induced stomatal closure. Concurrently, transcriptomic profiling and metabolic analyses further demonstrated that SlERF.D2 repressed the anthocyanin biosynthetic pathway, leading to a reduced anthocyanin content and increased reactive oxygen species (ROS) levels. Our findings delineate a novel regulatory module wherein SlERF.D2 coordinates stomatal closure and ROS homeostasis to modulate the sensitivity of tomato plant to osmotic stresses, providing an applicable target for improving osmotic stress adaptation in tomato.

## Introduction

Plants encounter diverse environmental challenges during their life cycles, among which osmotic stresses, such as drought and salt critically impair their growth and developmental processes [1]. These abiotic stressors induce multifaceted physiological responses, including ionic imbalance, ROS overproduction, osmotic changes, and hormonal modulation [2]. To mitigate these adverse effects, plants have evolved sophisticated adaptive strategies involving stomatal movement, ROS detoxification, and senescence initiation [3, 4]. Elucidating the signaling cascades regulating these stress responses facilitate the breeding of crop with improved adaptability to environmental stresses.

As a master regulator in plant osmotic stress adaptation, ABA orchestrates physiological adjustments from stomatal aperture modulation to transcriptome remodeling [5]. The ABA signaling cascade is composed of three core components: PYR/PYL/RCAR receptors, protein phosphatase 2Cs (PP2Cs), and SNF1-related protein kinase 2 s (SnRK2s) [6]. Within this network, PP2Cs act as critical molecular inhibitors by constitutively repressing ABA responses through SnRK2 dephosphorylation under non-ABA conditions [7, 8]. Genetic evidence indicates that PP2Cs play a suppressive role in stomatal closure triggered by osmotic stresses across multiple plant species, including Arabidopsis [9, 10], tomato [11], and rice [12]. Intriguingly, recent studies highlight a counteractive role of ethylene, a canonical senescence-promoting hormone, in ABA-mediated stomatal regulation [13]. Genetic analyses reveal an inverse regulatory relationship wherein ethylene-overproducing mutants exhibit impaired ABA-induced stomatal closure [14], whereas ethylene-insensitive lines display heightened sensitivity to ABA [15]. Mechanistic investigations suggest that ethylene-induced accumulation of antioxidant metabolites, particularly flavanols and ascorbic acid, may attenuate ABA signaling through scavenging ROS, which serve as key secondary messengers in stomatal movement [16, 17]. Nevertheless, whether ethylene modulates core ABA signaling components to inhibit ABA-mediated stomatal closure remains unresolved.

ROS are traditionally regarded as deleterious oxidants, exerting dual roles in plant stress responses: causing macromolecular damage to proteins, lipids, and nucleic acids under uncontrolled accumulation, while simultaneously functioning as essential signaling molecules during stress adaptation [18]. Beyond the primary osmotic stress imposed by drought and salt, an oxidative stress emerges when ROS generation overwhelms cellular antioxidant capacity [5]. The evolutionary conserved antioxidant system, encompassing enzymatic and non-enzymatic components, has been well characterized in conferring tolerance against abiotic stressors including drought and salt [19]. Notably, emerging evidence positions anthocyanin as a multifunctional phytoprotectant, exhibiting ROS-scavenging activity and membrane-stabilizing properties under diverse abiotic challenges [20, 21]. The biosynthesis of anthocyanin is governed by a sophisticated transcriptional regulatory network involving MYB-bHLH-WD40 ternary complexes, which integrate environmental cues to modulate anthocyanin accumulation [22–24]. Despite extensive documentation of stress-induced anthocyanin biosynthesis across plant species, the precise molecular mechanisms orchestrating this phenomenon remain incompletely elucidated.

AP2/ERF transcription factors, harboring a canonical AP2/ERF domain essential for sequence-specific DNA binding, has emerged as central regulatory nodes in plant abiotic stress adaptation [25]. Numerous ERF members have been functionally implicated in osmotic stress adaptation across diverse plant species. In Arabidopsis, overexpression of *AtERF1* confers enhanced drought resistance through accelerating stomatal closure [26], while AtERF34 negatively regulates leaf senescence, thereby improving salt stress adaptation [27]. Additionally, AtERF98 increases salt stress adaptation via transcriptionally upregulating the biosynthesis of ascorbic acid [28]. Beyond model systems, ERF homologs in crop species exhibit conserved stress regulatory roles: tobacco NtERF172 directly targets the promoter of *NtCAT*, thereby conferring enhanced drought adaptation [29]. In tomato, *SlERF5* overexpression results in heightened adaptation to osmotic stresses [30], whereas SlERF.B1 negatively regulates osmotic stress adaptation [31]. Notably, MdERF38 in apple (*Malus domestica*) coordinates drought-induced anthocyanin accumulation, linking secondary metabolism with stress adaptation [24]. Collectively, ERF proteins exhibit functional diversity in orchestrating plant osmotic stress responses. Despite these advances, critical knowledge gaps persist. Firstly, the mechanistic contributions of specific ERF clades to osmotic stress adaptation remain poorly resolved. Secondly, the downstream target genes through which ERFs mediate osmotic stress adaptation are largely uncharacterized, limiting our ability to construct comprehensive regulatory networks.

Tomato, a globally pivotal horticultural crop, faces severe limitations in productivity and fruit quality from abiotic stressors, particularly soil salinity and drought [25]. Here, we systematically characterized the involvement of SlERF.D2 in tomato osmotic stress adaptation. Quantitative expression profiling revealed that *SlERF.D2* transcript levels were upregulated under osmotic stresses and exogenous phytohormone treatments. CRISPR/Cas9-mediated knockout lines exhibited accelerated stomatal closure, elevated anthocyanin accumulation, and reduced ROS levels, culminating in enhanced drought and salt stress adaptation. Conversely, *SlERF.D2* overexpression lines displayed hypersensitivity to the osmotic stress. Mechanistic investigations demonstrated that *SlERF.D2* expression was upregulated by ethylene through the direct activation by SlEIL1/2/3/4. Meanwhile, SlERF.D2 directly targeted the *SlPP2C1* promoter and activated its transcription, thereby mediating ethylene-ABA crosstalk in stomatal regulation. Collectively, our findings establish SlERF.D2 as a negative modulator of tomato osmotic stress adaptation, operating predominantly through suppressing stomatal closure and destroying ROS homeostasis.

## Results

### Phylogenetic analysis, expression patterns, and subcellular localization of SlERF.D2

**Figure 1:**
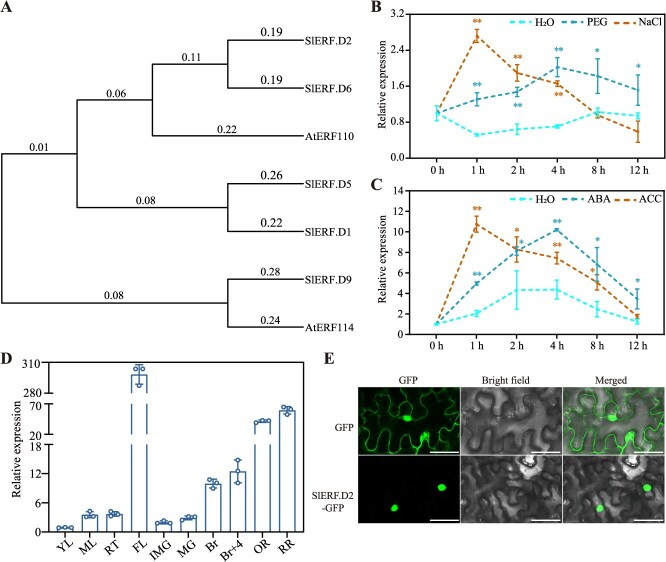
*SlERF.D2* expression patterns under osmotic stresses and hormone treatments. (A) Phylogenetic analysis of SlERF.D2 (NM_001346061.2) and related ERF homologs from *Solanum lycopersicum* (SlERF.D1, XM_004237435.5; SlERF.D5, XM_004237096.5; SlERF.D6, XM_004237769.5; SlERF.D9, XM_004242144.5) and *Arabidopsis thaliana* (AtERF110, NM_001344871.1; AtERF114, NM_125582.3). Phylogenetic reconstruction was performed in MEGA11 using the neighbor-joining method. Multiple sequence alignment was performed via BLASTP (NCBI). (B) Time-dependent transcriptional regulation of *SlERF.D2* in wild-type (WT) plants under osmotic stress induced by 10% (w/v) PEG6000 or 300 mM NaCl treatment. (C) Relative expression of *SlERF.D2* in detached leaves of WT plants following treatment with 200 μM ABA or ACC. (D) Spatiotemporal expression patterns of *SlERF.D2* across tissues of WT plants. Vegetative tissues: Young leaves (YL), Mature leaves (ML), Roots (RT); Reproductive tissues: Flowers (FL); Fruits: Immature green stage (IMG), Mature green stage (MG), Breaker stage (Br), 4 days post breaker stage (Br + 4), Orange ripe stage (OR), Red ripe stage (RR). (E) Cellular distribution of SlERF.D2-GFP chimeric protein in *Nicotiana benthamiana* leaves. Transient expression was achieved through *Agrobacterium tumefaciens*-mediated transformation. GFP fluorescence and bright-field microscopy revealed nuclear localization and cellular architecture. Scale bar = 50 μm. For (B)–(D), expression values of *SlERF.D2* gene are calculated using *SlActin7* as the reference gene and normalized to the expression level in WT plants at 0 h (B, C) or in YL (D). Data are means ± SD (*n* = 3). For (B) and (C), *t*-test (^*^P < 0.05, ^**^  *P* < 0.01).

To clarify the diverse functionality of key ERF transcription factors involved in the osmotic stress adaptation, a comprehensive analysis was performed on transcriptome datasets derived from prior investigation [32]. The comparative profiling with stringent thresholds (|Log_2_ fold change| ≥ 1, *P* < 0.05) revealed that *SlERF.D2* exhibited pronounced transcriptional activation under both drought and salt conditions, displaying co-expression patterns with the confirmed osmotic stress-responsive regulator *SlERF.D6* (also known as *SlERF84*) [25] (Fig. S1). Phylogenetic reconstruction based on ERF protein sequences from Arabidopsis and tomato further demonstrated that SlERF.D2, a 367-amino acid protein harboring a conserved AP2 DNA-binding domain (K160–N219), clustered closely with SlERF.D6 (Fig. 1A; Fig. S2). Consistent with its transcriptional co-regulation network under osmotic stresses, quantitative PCR assays indicated that an exposure to 10% PEG6000 (simulated drought stress) or 300 mM NaCl (salt stress) triggered a significant upregulation of *SlERF.D2* transcription (Fig. 1B). To dissect the hormonal regulation of *SlERF.D2* within the phytohormone-mediated stress signaling framework, we assessed its responsiveness to ABA and ethylene. Strikingly, exogenous treatment with 200 μM ABA or ACC (ethylene biosynthesis precursor) triggered *SlERF.D2* transcription, albeit with distinct kinetic profiles (Fig. 1C).

Tissue-specific expression profiling in *S. lycopersicum* cv. AC identified maximal *SlERF.D2* accumulation in floral tissues and fruits, with comparatively lower expression in roots and leaves (Fig. 1D). Transient transformation with the pro35S: SlERF.D2-GFP vector in *Nicotiana benthamiana* leaves demonstrated its nuclear targeting (Fig. 1E), consistent with its predicted function as a transcription factor. Collectively, these findings indicate that SlERF.D2 may assume a key modulator in osmotic stress adaptation via ABA and ethylene signaling pathways.

### SlERF.D2 negatively regulates tomato osmotic stress adaptation

To determine SlERF.D2’s role in regulating osmotic stress adaptation, *SlERF.D2*-knockout (CR) lines were created. Two sgRNA sequences were engineered to specifically recognize *SlERF.D2*'s second exon, with sgRNA1 being positioned upstream and sgRNA2 downstream of the predicted AP2 domain. Multiple mutations in *SlERF.D2* were confirmed using PCR and DNA sequencing methods. Specifically, *SlERF.D2*-CR#4 exhibited a homozygous 2-bp deletion while *SlERF.D2*-CR#6 harbored a homozygous 1-bp insertion at the sgRNA1 target site (Fig. S3A). The two mutations resulted in premature stop codons at amino acids 155 and 156 respectively, truncating the protein before the AP2 domain; both of the created mutants (CR#4 and CR#6) were used in the following analyses. Additionally, multiple independent *SlERF.D2*-overexpressing (OE) lines were also successfully generated and validated through RT-qPCR and western blotting, and two of the OE lines (OE#3 and OE#11) were used for subsequent studies (Fig. S3B and C).

**Figure 2:**
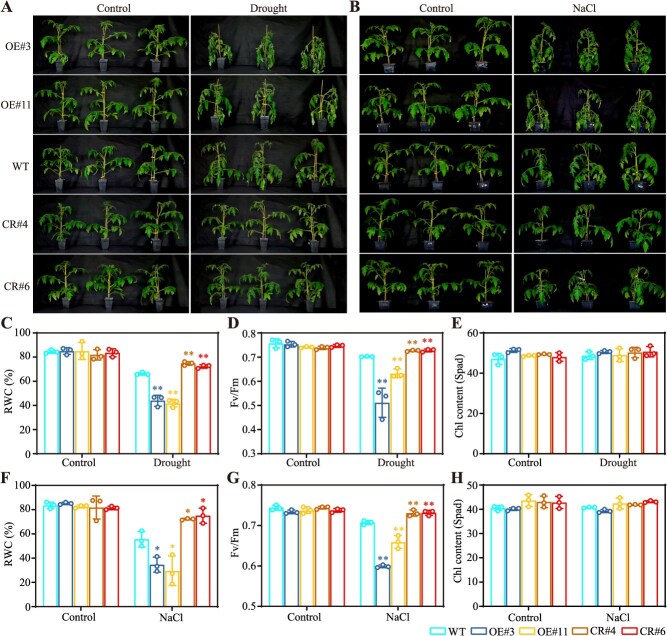
Phenotypes of *SlERF.D2* transgenic lines subjected to osmotic stresses. (A, B) Phenotypic changes of 42-day-old *SlERF.D2*-overexpressing (OE#3 and OE#11), and CRISPR-mediated knockout (CR#4 and CR#6) lines, in comparison to that of wild-type plants (WT), following a 5-day drought treatment (A) or a 3-day NaCl treatment (B). (C, F) Relative water contents (RWC) in the third true leaves after drought (C) and NaCl (F) treatments. (D, G) Photochemical efficiencies (Fv/Fm ratios) measured in the third true leaves after drought (D) and NaCl (G) treatments. (E, H) Chlorophyll contents quantification in the third true leaves after drought (E) and NaCl (H) treatments. Data are means ± SD (*n* = 3). *t*-test (^*^P < 0.05, ^**^  *P* < 0.01).

The 42-day-old *SlERF.D2*-OE and *SlERF.D2*-CR lines were exposed to two distinct stress treatments: drought stress (5 days of water withholding) and salt stress (3 days of 300 mM NaCl irrigation). Notably, under both stress conditions, the OE lines displayed accelerated wilting phenotypes, whereas the CR lines manifested an enhanced stress adaptation, as evidenced by milder wilting symptoms compared to that of WT (wild-type) (Fig. 2A and B). Under drought or NaCl stress, leaf relative water content (RWC) in the OE lines decreased significantly compared to that in WT plants, whereas the CR lines exhibited significantly higher RWC than the WT control (Fig. 2C and F). Quantitative evaluation of photosynthetic efficiency, measured by Fv/Fm, revealed that the OE lines exhibited a marked decline relative to that in the WT; conversely, the CR lines exhibited significantly improved Fv/Fm ratios under both stress treatments (Fig. 2D and G). Although osmotic stresses are widely recognized to accelerate leaf senescence [33], chlorophyll levels showed no significant differences between WT and the *SlERF.D2* transgenic plants under water treatment or osmotic stresses (Fig. 2E and H). The expression levels of the key osmotic stress-response genes *SlAREB1* and *SlDREB2A* were also examined in *SlERF.D2* transgenic plants under osmotic stress conditions. No expression differences were found under non-stress condition; however, under drought or salt stress, the expression levels of *SlAREB1* and *SlDREB2A* were significantly induced, with lower expressions in the OE plants and higher expressions in the CR plants than those in WT plants (Fig. S4). Collectively, these findings demonstrate that SlERF.D2 negatively regulates osmotic stress adaptation in tomato.

**Figure 3:**
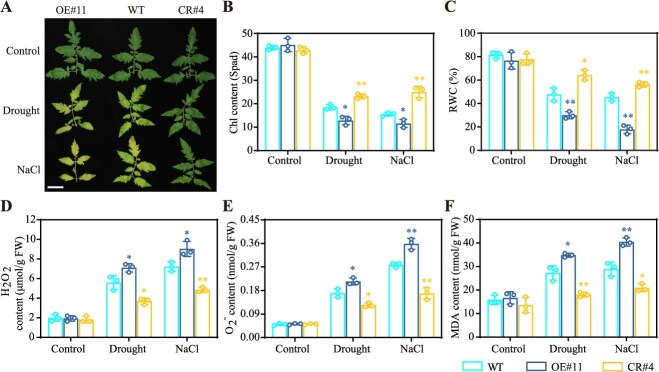
SlERF.D2 exacerbates oxidative damage and senescence progression induced by prolonged drought or salt stress in tomato. (A) Morphological symptoms in the third true leaves of 42-day-old WT, *SlERF.D2*-overexpressing (OE#11), and *SlERF.D2*-knockout (CR#4) plants exposed to 12-day drought or NaCl treatment. Scale bar = 5 cm. (B-C) Chlorophyll contents and relative water contents (RWC) after prolonged drought or salt stress. (D-F) Quantification of the oxidative stress markers in the leaf tissues: H_2_O_2_ contents (D), O_2_^−^ contents (E), and MDA contents (F). Data are means ± SD (*n* = 3). *t*-test (^*^P < 0.05, ^**^  *P* < 0.01).

Prolonged osmotic stresses trigger excessive ROS accumulation, also known as a hallmark of stress-induced leaf senescence. To assess ROS-induced damage, we subjected 42-day-old WT and *SlERF.D2* transgenic lines (OE#11 and CR#4) to prolonged drought or NaCl treatment (12 days), with phenotypic and biochemical analyses being carried out on the third true leaves. The OE#11 line exhibited a pronounced wilting and chlorosis, accompanied by significantly reduced chlorophyll content and RWC compared to those in the WT; conversely, the CR#4 line demonstrated an enhanced stress adaptation, with significantly higher chlorophyll retention and water status maintenance (Fig. 3A–C). Following the osmotic stress treatments, all genetic materials showed elevated H_2_O_2_ and O_2_^−^ levels, with the OE#11 line accumulating higher and the CR#4 line lower ROS concentrations than those in the WT (Fig. 3D and E). This differential oxidative stress responses corresponded with elevated malondialdehyde (MDA) contents, wherein the OE#11 line accumulated more MDA than WT, while the CR#4 line demonstrated a reduced MDA level compared to WT (Fig. 3F). These data collectively indicate that SlERF.D2 exacerbates oxidative damage and senescence progression under prolonged osmotic stresses.

### SlERF.D2 negatively regulates osmotic stress adaptation by suppressing stomatal closure

**Figure 4:**
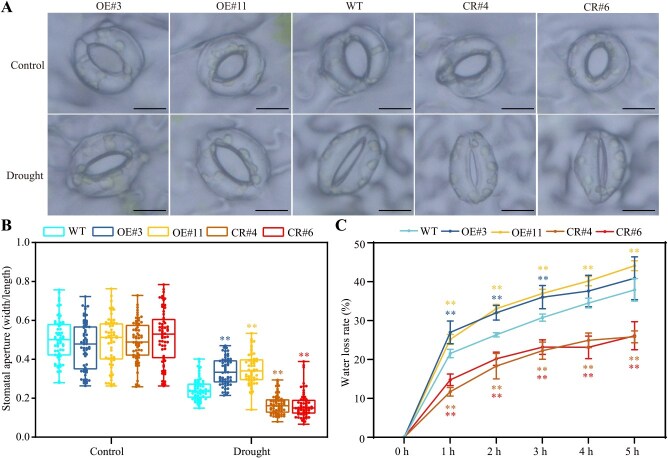
Regulatory role of SlERF.D2 in drought-induced stomatal closure. (A) Representative confocal micrographs illustrating stomatal morphology in the third true leaves of 42-day-old WT, *SlERF.D2*-overexpressing (OE#3 and OE#11), and knockout (CR#4 and CR#6) plants following 5-day drought stress. Scale bar = 10 μm. (B) Measurement of stomatal aperture (width/length ratios) under drought stress. Data are means ± SD (60 stomata per genotype from three biological replicates). (C) Leaf water loss kinetics of excised leaves incubated at 25°C/65% relative humidity (RH). Measurements were recorded at indicated time points. Data are means ± SD (*n* = 5). For (B) and (C), *t*-test (^**^  *P* < 0.01).

In response to osmotic stresses, stomatal closure is a critical movement implicating physiological changes, reducing transpirational water loss, and maintaining cellular osmotic homeostasis. To functionally characterize the relationship between altered osmotic stress adaptation and stomatal movement in the *SlERF.D2* transgenic plants, we performed quantitative analyses of stomatal aperture (quantified as width/length ratios) in 42-day-old WT and the *SlERF.D2* transgenic plants following drought (5 days) or NaCl (3 days) treatment. Under non-stress conditions, there were no significant differences between WT and the *SlERF.D2* transgenic plants. By contrast, after drought or NaCl treatment, the OE lines exhibited significantly impaired stomatal closure, while the CR lines displayed enhanced stomatal closure capacity relative to WT (Fig. 4A and B; Fig. S5A and B). Gravimetric analysis of excised leaf water loss validated the phenotypic variation, revealing a significantly enhanced water loss rate in the OE lines and attenuated dehydration in the CR lines relative to that in the WT (Fig. 4C). These results indicate that SlERF.D2 has a negative regulatory effect on stomatal closure and water retention under osmotic stresses.

### SlERF.D2 mediates the antagonism between ethylene and ABA signaling in stomatal closure process

**Figure 5:**
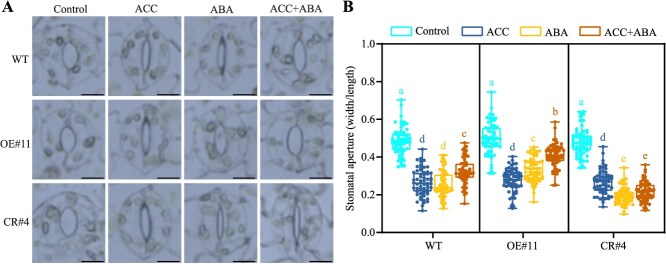
Ethylene suppresses ABA-induced stomatal closure in tomato. (A) Confocal micrographs demonstrating stomatal morphology in the third true leaves of 42-day-old WT, *SlERF.D2*-overexpressing (OE#11), and *SlERF.D2*-knockout (CR#4) plants under hormone treatments: 50 μM ACC (ethylene precursor), 10 μM ABA, and 10 μM ABA +50 μM ACC. Scale bar = 10 μm. (B) Stomatal aperture quantifications (width/length ratios) after individual or combined hormone treatments. Data are means ± SD (60 stomata per genotype from three biological replicates). Lowercase letters denote statistically distinct groups (two-way ANOVA followed by Tukey’s HSD post hoc test; *P* < 0.05).

Given that ABA and ethylene play critical roles in stress-triggered stomatal closure [34–36], and *SlERF.D2* expression levels were markedly upregulated in response to exogenous ABA or ACC treatment (Fig. 1C), we next investigated potential involvement of SlERF.D2 in ABA/ethylene-regulated stomatal movement. Although ACC treatment induced stomatal closure in tomato leaves compared to the control treatment, no significant differences in stomatal aperture were observed between *SlERF.D2* transgenic lines and WT plants following ACC treatment (Fig. 5). However, the stomatal closure induced by ABA was significantly suppressed in the *SlERF.D2*-OE line (OE#11) but markedly accelerated in the *SlERF.D2*-CR line (CR#4) compared to that in WT. These observations indicate that SlERF.D2 suppresses ABA-induced stomatal closure. Previous studies have reported that ethylene could antagonize ABA-induced stomatal closure [13]. To further investigate whether SlERF.D2 contributes to ethylene-ABA crosstalk during stomatal movement, the third true leaves of 42-day-old plants were also subjected to 10 μM ABA +50 μM ACC treatment. Assessments of stomatal aperture changes revealed that ethylene antagonized ABA-mediated stomatal closure, as evidenced by significantly larger stomatal apertures under the ABA + ACC treatment compared to ABA treatment alone. Furthermore, under the ABA + ACC treatment, the OE plants showed significantly larger stomatal apertures compared to the WT plants. Intriguingly, this ethylene-mediated antagonism was abolished in the CR plants, where ACC failed to reverse ABA-induced stomatal closure (Fig. 5). These findings collectively establish SlERF.D2 as an essential mediator of the ethylene-ABA signaling antagonism in stomatal closure.

**Figure 6:**
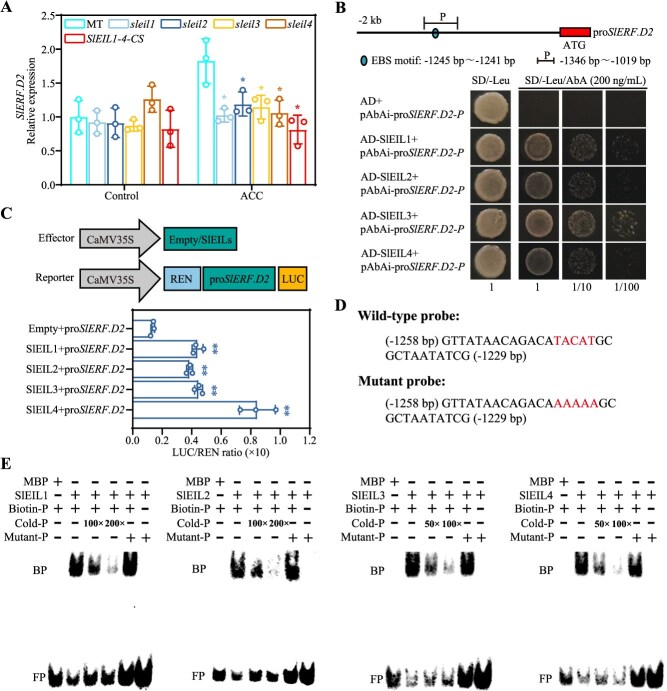
SlEIL1/2/3/4 activate the transcription of *SlERF.D2* by targeting its promoter. (A) Transcript abundance of *SlERF.D2* in the leaves excised from 35-day-old Micro-Tom (MT), *sleil1*, *sleil2*, *sleil3*, *sleil4* and *SlEIL1-4* co-suppression (*SlEIL1-4*-*CS*) lines after 4-h 200 μM ACC treatment. Expression values of *SlERF.D2* gene are calculated using *SlActin7* as the reference gene and normalized to its expression level in MT plants under control condition. (B) Binding of SlEIL1/2/3/4 to the *SlERF.D2* promoter. Upper panel: Schematic diagram of *SlERF.D2* promoter region (‘P’ indicates the promoter sequence fragment cloned into pAbAi bait vector, −1346 to −1019 bp). Lower panel: Y1HGold yeast harbored both pAbAi-pro*SlERF.D2* and AD-SlEIL1/2/3/4 vectors grown on synthetic dropout medium lacking leucine, in the presence or absence of 200 ng/ml aureobasidin A (AbA). (C) Transcriptional regulation of *SlERF.D2* by SlEIL1/2/3/4 through transient assays. Upper panel: Diagram of the plasmid employed in the dual-luciferase assay. Lower panel: firefly/renilla luciferase (LUC/REN) activity ratios in *Nicotiana benthamiana* leaves co-infiltrated with effector (35S: empty/35S: SlEILs) and reporter vector. (D) Sequences of probe (−1258 to −1229 bp) containing the EBS motif (TACAT) from the *SlERF.D2* promoter used in EMSA. (E) EMSA assays showing the interaction between SlEIL1/2/3/4 and the EBS motif in the *SlERF.D2* promoter. BP, bound probe. FP, free probe. ‘50×’, ‘100×’, and ‘200×’ indicate increasing amounts of Cold-P (unlabeled wild-type probe) used for competition. The amounts of Mutant-P (biotin-labeled mutant probe) were 20-fold those of the Biotin-P (biotin-labeled wild-type probe). For (A) and (C), data are means ± SD (*n* = 3). *t*-test (^*^P < 0.05, ^**^  *P* < 0.01).

**Figure 7:**
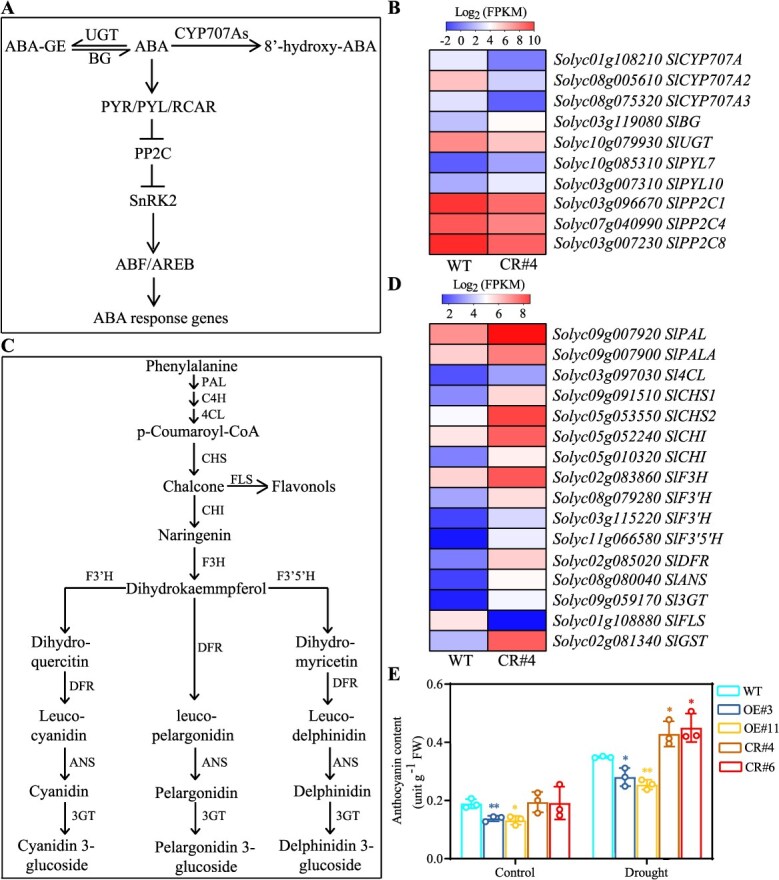
Knockout of *SlERF.D2* alters ABA metabolism, signaling, and anthocyanin biosynthesis under drought stress. (A) Schematic of core ABA metabolism and signaling pathway in tomato leaves. (B) Heatmap of ABA-related DEGs between WT and CR#4 following 5-day drought treatment. Color scale: Log_2_ (FPKM). (C) Schematic of the anthocyanin production route in tomato leaves. (D) Heatmap of DEGs in anthocyanin biosynthesis and metabolism between WT and CR#4 after 5-day drought treatment. Color scale: Log_2_ (FPKM). RNA-seq data for WT and CR#4 plants under drought condition were derived from three biological replicates each genotype. (E) Anthocyanin contents in the third true leaves of WT, *SlERF.D2*-overexpressing (OE#3 and OE#11), and *SlERF.D2*-knockout (CR#4 and CR#6) plants subjected to 5-day drought stress. Data are means ± SD (*n* = 3). *t*-test (^*^P < 0.05, ^**^  *P* < 0.01).

### Ethylene-induced *SlERF.D2* expression requires a direct activation by SlEIL1/2/3/4

The EIN3/EIL family, functioning as the core components of the ethylene signaling pathway, regulates ethylene-responsive genes expression and ethylene-mediated stomatal movement [34, 37]. To investigate whether the induction of *SlERF.D2* expression by ethylene is dependent on EIN3/EIL, we quantified *SlERF.D2* transcript levels in the leaves of the following genotypes after ACC treatment: MT (Micro-Tom, wild-type), *sleil1*, *sleil2*, *sleil3*, and *sleil4* single mutants, and *SlEIL1*–*4*-*CS* (*SlEIL1–4* co-suppression) plants. Compared with MT plants, ACC-induced *SlERF.D2* expressions were decreased in all *sleil* single mutants and *SlEIL1*–*4*-*CS* plants (Fig. 6A), indicating that *SlERF.D2* likely acted downstream of SlEIL1/2/3/4. Bioinformatic analysis of the *SlERF.D2* promoter identified a conserved EIN3-binding site (EBS) (Fig. 6B, upper panel), a known binding motif for EIN3/EIL family. Yeast one-hybrid (Y1H) assays under selective conditions (200 ng/ml AbA) confirmed physical interaction between SlEIL1/2/3/4 and *SlERF.D2* promoter, as demonstrated by colony formation (Fig. 6B, lower panel). Dual-luciferase reporter assays in *Nicotiana benthamiana* leaves co-transfected with CaMV35S-SlEIL1/2/3/4 and pro*SlERF.D2*-LUC constructs showed significantly enhanced LUC/REN ratios compared with CaMV35S-empty and pro*SlERF.D2*-LUC constructs, confirming that SlEIL1/2/3/4 activated the transcription of *SlERF.D2* (Fig. 6C). Electrophoretic mobility shift assays (EMSA) established a direct binding specificity. Recombinant MBP–SlEIL1/2/3/4 fusion proteins formed stable complexes with biotin-labeled *SlERF.D2* promoter fragments, as evidenced by distinct mobility shifts. Specific binding was confirmed through negative control with MBP protein alone, as well as competitive displacement with unlabeled probe and labeled mutant probe (Fig. 6D and E). Collectively, these results reveal that SlEIL1/2/3/4 directly target the EBS motif within the *SlERF.D2* promoter to enhance its transcription.

### Knockout of *SlERF.D2* enhances ABA signaling and anthocyanin biosynthesis

To uncover the molecular pathways through which SlERF.D2 regulates osmotic stress adaptation, RNA sequencing was performed on the third true leaves of 42-day-old WT and *SlERF.D2*-knockout (CR#4) plants exposed to the drought condition. Our analysis identified 3142 differentially expressed genes (DEGs) (|Log_2_ fold change| ≥ 1, *P* < 0.05), including 1906 transcriptionally activated and 1236 suppressed genes (Fig. S6). Given the functional role of SlERF.D2 in stomatal movement under osmotic stresses, DEGs associated with ABA metabolism and signaling were prioritized for further analysis. Transcript levels of genes involved in ABA catabolism (*SlCYP707A*, *SlCYP707A2*, *SlCYP707A3*, and *SlUGT*) exhibited significantly downregulated. By contrast, *SlBG*, which converts inactive ABA into its active form, exhibited a marked upregulation. Concurrently, the ABA receptor genes *SlPYL7* and *SlPYL10* showed significantly increased expressions. Conversely, the genes that negatively regulate ABA signaling (*SlPP2C1*, *SlPP2C4*, and *SlPP2C8*) exhibited substantial down-regulation (Fig. 7A and B). These coordinated transcriptional changes substantiate a pivotal role of SlERF.D2 in modulating ABA pathway. Further analysis of the transcriptome identified the differential expressions of 16 genes involved in anthocyanin synthesis (Fig. 7C and D). Given the established role of anthocyanin in ROS scavenging, we quantified anthocyanin content under drought or salt treatment. It was revealed that the anthocyanin accumulation decreased significantly in the OE lines but increased in the CR lines compared to that in WT under drought or salt treatment (Fig. 7E; Fig. S5D). These findings collectively demonstrate that SlERF.D2 negatively regulates ABA signaling and anthocyanin biosynthesis, thereby impairing stomatal closure and reducing ROS detoxification capacity under osmotic stresses.

### SlPP2C1 is involved in the antagonism between ethylene and ABA signaling mediated by SlERF.D2 in regulating stomatal movement

**Figure 8:**
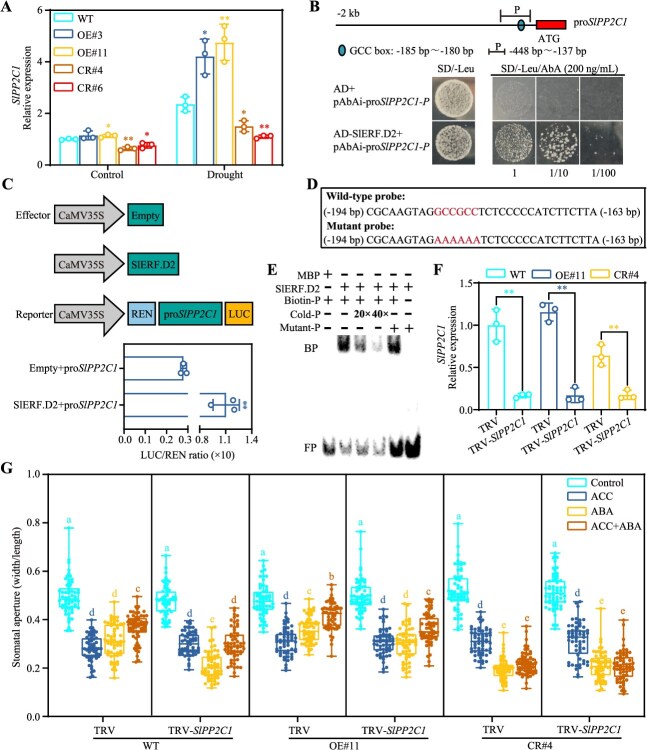
SlERF.D2 promotes the transcription of *SlPP2C1* by targeting its promoter. (A) Transcript abundance of *SlPP2C1* in the third true leaves of 42-day-old WT and *SlERF.D2* transgenic plants after 5-day drought stress. Expression values of *SlPP2C1* gene are calculated using *SlActin7* as the reference gene and normalized to the expression level in WT plants under control condition. (B) Binding of SlERF.D2 to the *SlPP2C1* promoter. Upper panel: Schematic of *SlPP2C1* promoter region (‘P’ indicates the sequence fragment cloned into pAbAi bait vector, −448 to −137 bp). Lower panel: Y1HGold yeast harbored both pAbAi-pro*SlPP2C1* and AD-SlERF.D2 vectors grown on synthetic dropout medium lacking leucine, in the presence or absence of 200 ng/ml aureobasidin A (AbA). (C) Transcriptional regulation of *SlPP2C1* by SlERF.D2 through transient assays. Upper panel: Diagram of the plasmid employed in the dual-luciferase assay. Lower panel: firefly/Renilla luciferase (LUC/REN) activity ratios in *Nicotiana benthamiana* leaves co-infiltrated with the effector (35S: empty/35S: SlERF.D2) and the reporter vector. (D) Sequences of probe (−194 to −163 bp) containing the GCC box (GCCGCC) from the *SlPP2C1* promoter used in EMSA. (E) EMSA assays showing the interaction between SlERF.D2 and the GCC box in the *SlPP2C1* promoter. BP, bound probe. FP, free probe. ‘20×’ and ‘40×’ indicate increasing amounts of Cold-P (unlabeled wild-type probe) for competition. The amounts of Mutant-P (biotin-labeled mutant probe) were 20-fold those of the Biotin-P (biotin-labeled wild-type probe). (F) Transcript abundance of *SlPP2C1* in the third true leaves of VIGS plants. Expression values of *SlPP2C1* gene are calculated using *SlActin7* as the reference gene and normalized to the expression level in WT-TRV plants. (G) Stomatal aperture quantifications (width/length ratios) of VIGS plants under hormone treatments: 50 μM ACC, 10 μM ABA, and 10 μM ABA +50 μM ACC. For (A), (C), and (F), data are means ± SD (*n* = 3). *t*-test (^*^P < 0.05, ^**^  *P* < 0.01). For (G), data are means ± SD (60 stomata per genotype from three biological replicates). Lowercase letters denote statistically distinct groups (two-way ANOVA followed by Tukey’s HSD post hoc test; *P* < 0.05).

An integrated analysis of RNA-seq data identified SlERF.D2 as a putative transcriptional regulator of *SlPP2C1* (Fig. 7B), a pivotal regulator in the ABA signal transduction cascade known to negatively modulate ABA-mediated fruit ripening and drought stress responses [38, 39]. RT-qPCR validated that *SlPP2C1* expression levels were induced in both WT and the *SlERF.D2* transgenic plants under osmotic stresses. Notably, under normal and osmotic stress conditions, *SlPP2C1* expression levels showed significant increases in the OE lines and decreases in the CR lines compared to those in the WT (Fig. 8A; Fig. S5C). This transcriptional regulation pattern establishes *SlPP2C1* as a potential downstream target of SlERF.D2. A bioinformatic analysis of *SlPP2C1* promoter sequences identified a conserved GCC box cis-element (Fig. 8B, upper panel), a known binding motif for AP2/ERF transcription factors. Y1H assays under selective conditions (200 ng/ml AbA) confirmed a physical interaction between SlERF.D2 and the *SlPP2C1* promoter, as evidenced by colony formation (Fig. 8B, lower panel). Functional validation via dual-luciferase reporter assays demonstrated a significant enhancement of LUC/REN ratio when *Nicotiana benthamiana* leaves were transfected with CaMV35S-SlERF.D2 and pro*SlPP2C1*-LUC constructs, confirming that SlERF.D2 activated the expression of *SlPP2C1* (Fig. 8C). To establish direct binding specificity, EMSA assays were conducted. Recombinant MBP–SlERF.D2 fusion protein formed stable complexes with biotin-labeled *SlPP2C1* promoter fragments, as evidenced by distinct mobility shifts. Binding specificity was confirmed through negative control with MBP protein alone, as well as competitive displacement with unlabeled probe and labeled mutant probe (Fig. 8D and E). These findings collectively reveal that SlERF.D2 targets the GCC box motif in *SlPP2C1* promoter to enhance its transcription.

To investigate whether *SlPP2C1* expression could be induced by ethylene, WT plants and *SlERF.D2* transgenic lines were treated with ACC. Results showed that ACC treatment induced *SlPP2C1* expression, and this induction was compromised in the CR#4 line but enhanced in the OE#11 line (Fig. S7). Similarly, ABA treatment also induced *SlPP2C1* expression. However, under ABA treatment alone, *SlPP2C1* expression levels in the *SlERF.D2* transgenic lines showed no significant difference compared to those in WT. Following combined treatment with ABA and ACC, *SlPP2C1* expression levels in WT plants and OE#11 line were significantly higher than those under ABA treatment alone. In contrast, *SlPP2C1* expression levels in the CR#4 line remained comparable to those observed under ABA treatment alone. Collectively, these results indicate that SlERF.D2 is required for the ethylene-mediated induction of *SlPP2C1* expression, but does not significantly influence the ABA-induced expression of *SlPP2C1*. Next, to define the role of SlPP2C1 within the SlERF.D2-mediated antagonism between ethylene and ABA signaling in regulating stomatal movement, *SlPP2C1* was silenced using virus-induced gene silencing (VIGS) in WT plants and *SlERF.D2* transgenic lines (Fig. 8F). *SlPP2C1* silencing did not affect ACC-induced stomatal closure (Fig. 8G). Under ABA treatment, *SlPP2C1* suppression significantly enhanced ABA-induced stomatal closure in both WT plants and OE#11 line compared to TRV controls. Under co-treatment with ACC and ABA, *SlPP2C1*-silenced WT plants and OE#11 line exhibited significantly larger stomatal apertures than those under ABA treatment alone. However, under co-treatment, the stomatal apertures of *SlPP2C1*-silenced WT plants and OE#11 line still maintained significantly smaller than those of TRV controls. These results indicate that *SlPP2C1* silencing could attenuate but not fully abolish the inhibitory effect of ACC on ABA-induced stomatal closure. In the CR#4 line, *SlPP2C1* silencing had no significant effect on stomatal aperture under either ABA alone or ACC + ABA co-treatment compared to TRV controls. Collectively, these results demonstrate that SlPP2C1 suppresses ABA-induced stomatal closure and functions in the SlERF.D2-dependent pathway that mediates the ethylene-ABA antagonism during stomatal regulation.

## Discussion

The AP2/ERF superfamily is crucial for plant responses to abiotic stresses [40]. Genome-wide analyses have identified 140 AP2/ERF members in tomato, which show functional duality in osmotic stress adaptation, acting as positive or negative regulators [41, 42]. For example, overexpression of *SlERF84* in Arabidopsis enhances osmotic stress tolerance [25], whereas overexpression of *SlERF.B1* in tomato reduces osmotic stress tolerance [31]. These contrasting observations underscore the complexity of ERF-mediated osmotic stress response mechanisms and the necessity for a precise elucidation of related signaling pathways and their regulation. Given the shared osmotic stress induced by drought and salt, comparative transcriptomic profiling under these conditions enables identification of core ERF regulators orchestrating osmotic stress adaptation. In this study, an integrated analysis of tomato drought- and salt-stress transcriptome data identified *SlERF.D2* as a central ERF transcription factor of drought and salt stresses, which exhibited co-expression patterns and evolutionary conservation with *SlERF.D6* (Fig. S1; Fig. 1A and B). Osmotic stresses trigger the accumulation of ethylene and ABA, phytohormones that regulate plant stress adaptation [43–47]. Our results showed that exogenous application of either ACC or ABA significantly upregulates the transcription of *SlERF.D2* in tomato (Fig. 1C), indicating that the induction of *SlERF.D2* under osmotic stresses is likely mediated by both ethylene and ABA. Paradoxically, despite its transcriptional activation by ethylene and ABA, SlERF.D2 functions as a negative regulator of osmotic stress adaptation in tomato. This phenomenon is consistent with observations for *SlERF.B1*, whose expression is increased under NaCl or mannitol treatment. However, *SlERF.B1* overexpression reduces tomato stress adaptation by suppressing the activity of ROS scavenging enzymes and stomatal closure [31]. Similarly, in soybean (*Glycine max*), *GmERF105* transcription is elevated under salt or dehydration stress but it negatively modulates osmotic stress tolerance by attenuating antioxidant capacity [48]. Such counterintuitive induction of negative regulators may reflect homeostatic feedback mechanisms to prevent metabolic exhaustion and oxidative damage caused by hyperactive stress responses.

RWC serves as a physiological index of water status in plant organs. The marked reduction in RWC observed under osmotic stresses reflects cellular dehydration, culminating in structural collapse and metabolic dysfunction [49]. Stomatal closure functions as a critical mediator of water conservation under stress conditions, being finely regulated by osmotic stresses and hormonal networks [18, 50, 51]. In this study, *SlERF.D2* overexpression increased sensitivity to osmotic stresses via accelerating water loss, while *SlERF.D2* knockout enhanced stress adaptation by delaying water loss (Fig. 2). Specifically, drought- or salt-induced stomatal closure was compromised in the OE plants but augmented in the CR plants (Fig. 4; Fig. S5A and B). These results indicate a regulatory involvement of SlERF.D2 in stomatal dynamics. This is reminiscent of phytohormones, which act as central regulators, with ABA exerting predominant control over stomatal movement [13]. Notably, we observed that ABA-mediated stomatal closure was attenuated in the OE plants but potentiated in the CR plants. Furthermore, we identified an antagonistic crosstalk between ABA and ethylene signaling, a phenomenon previously documented in various plant developmental processes and stress responses [13]. Intriguingly, ethylene-mediated suppression of ABA-triggered stomatal closure was abolished in the CR plants but exacerbated in the OE plants (Fig. 5). These observations establish SlERF.D2 as a critical regulatory node mediating ethylene-ABA signaling antagonism during stomatal closure. The EIN3/EIL transcription factors are essential for ethylene-induced fruit ripening and stomatal movement regulation through the core ethylene signaling pathway [34, 37, 52]. In this study, we observed that ethylene-induced *SlERF.D2* expression was significantly attenuated in *sleil1/2/3/4* single mutants and *SlEIL1–4* co-suppression line. SlEIL1/2/3/4 directly bound to the EBS motif in the *SlERF.D2* promoter to enhance *SlERF.D2* transcription (Fig. 6). These results indicate that ethylene-induced *SlERF.D2* expression occurs in an SlEIL1/2/3/4-dependent manner and provide the molecular basis for SlERF.D2’s role in the antagonism of ethylene-ABA signaling in regulating stomatal movement.

Osmotic stresses induce peroxidative cascades characterized by ROS hyperaccumulation, resulting in oxidative cellular injury, chlorophyll degradation, and premature leaf senescence [31]. To mitigate these deleterious effects, plants deploy a coordinated antioxidant defense system wherein anthocyanin serves as a critical modulator of redox homeostasis [53, 54]. Emerging evidence implicates ERF proteins in the transcriptional orchestration of anthocyanin biosynthesis, as exemplified by PpERF105-mediated transcriptional repression of *PpMYB140* that attenuates anthocyanin biosynthesis in pear [55]. Conversely, MdERF38-dependent transactivation of *MdMYB1* potentiates anthocyanin accumulation in the drought-stressed apple [24]. Our data demonstrated that *SlERF.D2* overexpression significantly suppressed anthocyanin accumulation and elevated ROS levels. By contrast, the knockout lines exhibited enhanced anthocyanin production and ROS scavenging capacity (Fig. 3D–F; Fig. 7D and E; Fig. S5D). These metabolic alterations demonstrated mechanistic links with senescence progression, as leaf senescence induced by prolonged osmotic stress conditions was accelerated in the OE lines, manifested through reduced chlorophyll content compared to that in WT, whereas the CR lines exhibited a contrasting phenotype (Fig. 3A and B). These data align with reports demonstrating enhanced osmotic stress adaptation in anthocyanin-enriched tomato genotypes [56]. Collectively, these findings establish SlERF.D2 as a negative regulator of osmotic stress adaptation via modulating anthocyanin production, ROS homeostasis, and leaf senescence.

Through rapid stomatal regulation, ABA functions as a central orchestrator of plant osmotic stress adaptation [33]. The crucial components of ABA signal transduction in tomato have been extensively characterized, with a particular emphasis on clade A PP2C phosphatases. Notably, *SlPP2C1* exhibits pronounced transcriptional upregulation in both fruits and leaves under dehydration and exogenous ABA treatments, functioning as a suppressor in ABA signal transduction under drought stress [38, 39, 57]. This regulatory paradigm extends to paralogs *SlPP2C2*, *SlPP2C3*, and *SlPP2C5*, whose expressions are similarly induced by ABA while exerting negative control over drought stress adaptation [11, 58, 59]. Paradoxically, while stress-induced ABA accumulation inactivates PP2Cs to derepress SnRK2 kinases and initiate ABA signal transduction, transcriptional upregulation of PP2C genes under ABA treatment establishes an autoregulatory circuit. This negative feedback mechanism fine-tunes ABA sensitivity by maintaining cellular homeostasis and desensitizing plants to prolonged ABA elevation [60]. Emerging evidence suggests that ERF proteins frequently mediate stress responses through ABA-dependent mechanisms. OsERF71 promotes drought adaptation through activating several ABA responsive genes and proline biosynthesis genes in rice [61], whereas ORA47 modulates water stress sensitivity through *ABI2* transcriptional regulation in Arabidopsis [62]. In this study, *SlPP2C1* expression was up-regulated by osmotic stresses and ethylene in an SlERF.D2-dependent manner, whereas ABA-induced expression of *SlPP2C1* was independent of SlERF.D2 (Fig. 8A; Fig. S5C; Fig. S7). Mechanistic analysis further revealed that SlERF.D2 targeted the GCC box motif in *SlPP2C1* promoter, enhancing its transcription (Fig. 8B–D). Genetic silencing of *SlPP2C1* not only enhanced ABA-induced stomatal closure but also compromised ethylene-mediated suppression of this process (Fig. 8G). These results establish SlERF.D2-mediated transcriptional activation of *SlPP2C1* as a central regulatory node integrating osmotic stresses, ABA, and ethylene signaling.

**Figure 9:**
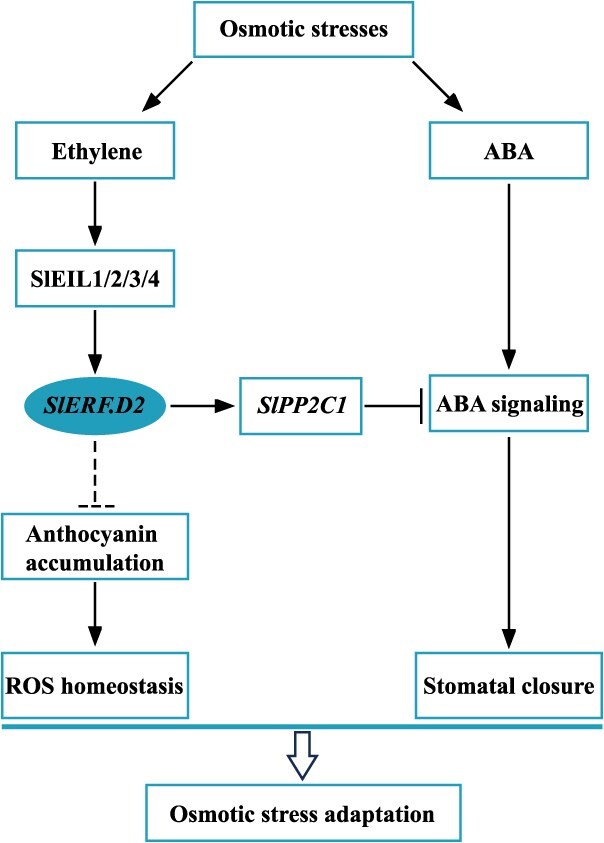
A proposed working model of SlERF.D2-mediated osmotic stress adaptation in tomato. *SlERF.D2* expression is induced by ethylene and is directly activated by SlEIL1/2/3/4, the core transcription factors in the ethylene signaling pathway. *SlPP2C1*, a negative regulator of ABA signaling that suppresses ABA-mediated stomatal closure, acts downstream of SlERF.D2 and is transcriptionally activated by SlERF.D2. Ethylene regulates *SlPP2C1* expression through this SlERF.D2-dependent mechanism. Consequently, SlERF.D2-mediated upregulation of *SlPP2C1* attenuates ABA signaling and ABA-induced stomatal closure, thereby mediating the antagonistic crosstalk between ethylene and ABA pathways in the regulation of stomatal movement. Concurrently, SlERF.D2 suppresses anthocyanin accumulation under osmotic stresses, suggesting its role in regulating reactive oxygen species (ROS) homeostasis through anthocyanin metabolism. Collectively, SlERF.D2 attenuates osmotic stress tolerance through dual mechanisms: (i) stomatal closure suppression and (ii) redox homeostasis disruption. However, direct regulation of anthocyanin biosynthesis genes by SlERF.D2 remains unresolved. Arrowheads indicate positive regulation; T-bars denote negative regulation; solid lines, mechanistically confirmed pathway; dashed lines, putative associations requiring further mechanistic characterization.

Taken together, SlERF.D2 compromises osmotic stress adaptation in tomato through dual regulatory mechanisms: (i) impairment of ABA-dependent stomatal closure via *SlPP2C1* activation, and (ii) suppression of anthocyanin-facilitated ROS detoxification pathways. Intriguingly, an antagonistic relationship exists between ethylene and ABA signaling, whereby ethylene suppresses ABA-induced stomatal closure through the SlEIL1/2/3/4–SlERF.D2–SlPP2C1 transcriptional cascade (Fig. 9). These revelations advance our understanding of ERF-mediated stress adaptation, and importantly, identify SlERF.D2 as a candidate ERF member applicable for engineering stress-adaptative crops. Subsequent research needs to concentrate on exploring the regulatory roles of SlERF.D2 involved in the anthocyanin biosynthesis and determining its interacting proteins within tomato osmotic stress response networks to refine the strategies for optimizing plant performance under osmotic stress.

## Materials and methods

### Plant materials and growth conditions

Tomato (*Solanum lycopersicum*) and tobacco (*Nicotiana benthamiana*) were cultivated in long-day conditions as described previously [63], with tomato Ailsa Craig (AC; LA2838A) being used as the WT control and genetic transformation material. The tomato cultivar Micro-Tom (MT), *sleil1*, *sleil2*, *sleil3*, *sleil4* and *SlEIL1–4* co-suppression (*SlEIL1–4*-*CS*) lines were obtained from Professor Hongwei Guo's laboratory (Department of Biology, Institute of Plant and Food Science, Southern University of Science and Technology, Shenzhen 518 055, China).

### Virus-induced gene silencing (VIGS) in tomato leaf

VIGS was performed using the pTRV system (pTRV1 and pTRV2 vectors) provided by Professor Xuequn Pang (College of Life Sciences, South China Agricultural University, Guangzhou, 510 642, China). To minimize potential off-target effects, a unique 400-bp cDNA fragment of *SlPP2C1* gene was designed using the online VIGS tool (http://solgenomics.net/tools/vigs). This fragment was cloned into the pTRV2 vector via homologous recombination. Subsequently, pTRV1 and the recombinant pTRV2 vector were separately introduced into *Agrobacterium tumefaciens* strain GV3101. Seven days after sowing, seedlings with fully expanded cotyledons were selected for injection. Plasmid construction and tomato leaf infiltration were performed according to a previous study [64]. The primer sequences used are provided in Table S1.

### Plant treatments

For *SlERF.D2* expression pattern analysis, all stress treatments were conducted on 42-day-old WT tomato plants grown under controlled conditions. (i) osmotic stresses: WT plants were root-drenched with 10% (w/v) PEG6000 or 300 mM NaCl solution for 12 h under continuous light. (ii) phytohormones: the third true leaves (counted acropetally from the stem base) from WT plants were excised and floated on 200 μM ABA or ACC solutions for 12 h under continuous light. Leaf samples were collected at 0, 1, 2, 4, 8, and 12 h, respectively.

For phenotypic analysis of plants subjected to osmotic stresses, all stress treatments were conducted on 42-day-old WT and *SlERF.D2* transgenic plants grown under controlled conditions. (i) drought stress: plants were withholding irrigation for 12 days to simulate progressive drought conditions. Photographs were taken and samples were collected on the 5th and 12th days after treatment. (ii) salt stress: plants were irrigated with 300 mM NaCl solution for 12 days. Photographs were taken and samples were collected on the 3th and 12th days after treatment.

For stomatal index analysis of plants subjected to hormone treatments, the third true leaves were excised from 42-day-old WT plants, *SlERF.D2* transgenic lines, and *SlPP2C1*-VIGS plants (35 days post-infiltration). To ensure full opening of leaf stomata, the excised leaves were pre-incubated in buffered medium under constant illumination at 25°C for 3 h. Then, leaves were treated with: (i) 50 μM ACC, (ii) 10 μM ABA, (iii) 10 μM ABA +50 μM ACC, and (iv) buffer (control) for 3 h. The buffer composition remained consistent with that described in previous studies [16]. After treatments, epidermal strips were collected for determination of the stomatal index.

### Vector construction and plant transformation

To generate *SlERF.D2* CRISPR knockout mutants, two single-guide RNAs (sgRNA1 and sgRNA2) targeting the second exon of the *SlERF.D2* were designed as reported earlier [65]. The sgRNAs were inserted into the pCBC-DT1T2 vector via PCR amplification and cloned into the pHEE401E-DsRed vector (*Bsa* I sites). To generate *SlERF.D2* overexpression, the complete coding sequence of *SlERF.D2* (NM_001346061.2) was inserted into the pCambia1306 vector without the termination codon, with a C-terminal 3 × Flag tag. Constructs transfer to *Agrobacterium* and subsequent *Agrobacterium*-driven transformation of tomato cotyledons followed the protocol described in a previous study [66]. For CRISPR/Cas9-based genetic modification, the genomic DNA segments adjacent to the target region were amplified, sequenced, and aligned with the coding sequence of *SlERF.D2*. The overexpression of *SlERF.D2* was confirmed in the transgenic lines by RT-qPCR. Homozygous knockout (T2) and overexpression (T3) lines were used for subsequent analysis. The primer sequences used are provided in Table S1.

### Subcellular localization

For subcellular localization analysis of SlERF.D2, the coding sequence was cloned into pCambia1302-GFP to express SlERF.D2-GFP fusion proteins. Transient expression in 30-day-old tobacco leaves was performed using the method described in a previous study [31]. GFP fluorescence observation was carried out as previously described [67]. The specific primers are listed in Table S1.

### Measurement of stomatal apertures

To investigate the closure response of stomatal apertures, the third true leaves of plants were selected for analysis. Abaxial epidermal strips were carefully excised using sharp-pointed tweezers and their stomatal morphology was immediately examined and visualized using a light microscope equipped (Nikon Eclipse Ni-U, 40 × objective) with a DP27 digital camera. Stomatal apertures were quantified via Photoshop software, with stomatal conductance determined by the width-to-length ratio. Three biological replicates (individual plants) per genotype were analyzed, with 20 stomatal measurements per leaf.

### Stress adaptation assays

To investigate physiological responses to osmotic stresses, 42-day-old WT and *SlERF.D2* transgenic plants were exposed to drought stress for 5 and 12 days or salt stress for 3 and 12 days. Key physiological parameters including leaf chlorophyll content, Fv/Fm ratio, relative water content (RWC), and anthocyanin content were subsequently quantified. (i) Chlorophyll contents were measured using a SPAD-502 PLUS chlorophyll meter as described previously [68]. (ii) Fv/Fm ratios were determined following the method outlined in a previous study [69]. (iii) Leaf RWCs were quantified as previously described [70] (iv) Contents of H_2_O_2_, O_2_^−^, and MDA were quantified spectrophotometrically using commercial assay kits (H_2_O_2_: H_2_O_2_–2-Y; O_2_^−^: SA-2-G; MDA: MDA-2-Y; Suzhou Comin Biotechnology Co., Ltd, China) following the manufacturer protocols. (v) Total anthocyanins were extracted and quantified using a TU-1900 UV–Vis spectrophotometer, following the method described in a previous study with modifications [71]. Briefly, frozen leaf tissue (0.1 g fresh weight) was pulverized in liquid nitrogen and homogenized in 1 ml of 1% (v/v) HCl/methanol. The homogenate was incubated in darkness at 4°C for 16 h, followed by centrifugation at 12 000 × g for 20 min. Absorbance of the supernatant was measured at 530, 620, and 650 nm using a UV–Vis spectrophotometer (TU-1900). Relative anthocyanin contents were calculated as: Anthocyanin Units = (*A*_530_ − *A*_620_) − 0.1 × (*A*_650_ − *A*_620_). One unit corresponds to 0.1 absorbance difference. (vi) To determine the water loss rate, the third true leaves of 42-day-old WT and *SlERF.D2* transgenic plants were excised and immediately weighed, designated as W_0_. Subsequently, the leaves were exposed to continuous illumination at 25°C with 60% relative humidity conditions for 5 h, with subsequent re-weighing at 1-h intervals, denoted as W_t_. The rate of water loss in the detached leaves was calculated using the formula: (W_0_-W_t_)/W_0_ × 100%. Five independent plants per line were selected, with one leaf chosen from each plant for analysis.

### RNA isolation and gene expression analysis

Leaf, root, flower, and fruit pericarp tissues were flash-frozen in liquid nitrogen. Reagents and methods for RNA extraction, reverse transcription, and gene expression analysis followed the descriptions in previous studies [68]. Transcript levels of *SlActin7* were used as the reference, with relative gene expression calculated by the 2^−ΔΔCt^ method. Primers used for qPCR are provided in Table S1.

### Protein extraction and western blotting

Leaf tissues (200 mg) harvested from WT and *SlERF.D2*-overexpressing plants were flash-frozen and homogenized. Total proteins were extracted following a modified protocol from prior studies [68]. The frozen homogenate was mixed with 800 μl protein extraction buffer and vortexed thoroughly. After 30-min ice incubation, lysate was spun at 10 000 × g for 20 min. Supernatant was mixed with 2 × loading buffer, boiled at 100°C for 10 min, and re-spun at 10 000 × g for 15 min to remove debris. Proteins were separated by 10% SDS-PAGE and immunoblotted with anti-Flag (Agrisera).

### RNA sequencing and analysis

For the transcriptomic profiling of the WT and *SlERF.D2*-knockout line (CR#4) under drought condition for 5 days, the third true leaves of plants were collected. Samples were immediately flash-frozen and stored at −80°C. Each genotype included three biological replicates. RNA sequencing was conducted on the Novaseq X Plus System by Shanghai Majorbio Biopharm Biotechnology Co., Ltd. HiSat2 (default settings) was used to map RNA-seq reads to the SL4.0 version of the Tomato Genome. DESeq2 was employed to screen DEGs under thresholds of |Log_2_ fold change| ≥ 1 and *P* < 0.05. Data preprocessing and downstream analysis were performed on the Majorbio Cloud Platform (www.majorbio.com). DEGs between WT and *SlERF.D2*-CR#4 under drought stress are provided in Table S2.

### Dual-luciferase assay

The full-length coding sequences of *SlERF.D2*, *SlEIL1*, *SlEIL2*, *SlEIL3*, and *SlEIL4* were individually cloned into the pCHF3 vector to generate effector vectors (pCHF3-SlERF.D2 and pCHF3-SlEIL1/2/3/4). The promoter fragments of *SlERF.D2* (−1346 to −1019 bp) and *SlPP2C1* (−448 to −137 bp) were PCR-amplified and individually cloned into the pGreenII 0800-LUC vector to generate reporter vectors (0800-pro*SlERF.D2* and 0800-pro*SlPP2C1*). Equal concentrations (OD = 0.6) of *Agrobacterium tumefaciens* GV3101 (pSoup-P19) carrying effector and reporter plasmids were co-introduced into 30-day-old tobacco leaves. Thirty six to forty eight hours post-injection, the samples were immediately frozen and stored at −80°C. Subsequent analytical methods were as described in a previous study [63]. Primers used for plasmid construction are provided in Table S1.

### Yeast one-hybrid assay (Y1H)

The Y1H assay was performed using the Matchmaker™ Gold Yeast One-Hybrid System (TaKaRa Bio, Beijing) following the manufacturer’s protocol. The full-length coding sequences of *SlERF.D2*, *SlEIL1*, *SlEIL2*, *SlEIL3*, and *SlEIL4* were individually cloned into the pGADT7 vector to generate prey vectors (AD-SlERF.D2 and AD-SlEIL1/2/3/4). The promoter fragments of *SlERF.D2* (−1346 to −1019 bp) and *SlPP2C1* (−448 to −137 bp) were PCR-amplified and individually cloned into the pAbAi vector to generate bait vectors (pAbAi-pro*SlERF.D2* and pAbAi-pro*SlPP2C1*). Following linearization, the bait plasmids were transferred to Y1H Gold yeast. The optimal AbA (aureobasidin A) concentration (200 ng/ml) was determined to suppress autonomous activation of the bait system. For interaction analysis, the prey construct (AD-SlERF.D2) and negative control (empty AD) were individually introduced into bait-harboring yeast cells. Transformants were selected using SD/−Leu plates (leucine-lacking synthetic dropout) with or without 200 ng/ml AbA and cultured at 30°C for 2 to 3 days. A growth on AbA-containing medium indicated the protein-DNA interaction. Primers used for plasmid construction are provided in Table S1.

### Electrophoretic mobility shift assay (EMSA)

The full-length coding sequences of *SlERF.D2*, *SlEIL1*, *SlEIL2*, *SlEIL3*, and *SlEIL4* were individually cloned into the pMAL-c5G vector via homologous recombination to generate MBP-fusion protein constructs. The pMAL-c5G vector was digested with *Sal* I and *Pst* I for *SlERF.D2* and *SlEIL1/2/3/4* genes' cloning. Recombinant MBP-SlERF.D2/SlEIL1/2/3/4 and MBP proteins were separately expressed in *Escherichia coli* Rosetta (DE3) strains, induced with 0.25 mM isopropyl β-d-1-thiogalactopyranoside (IPTG) at 16°C for 16 h. For the investigation of specific protein-DNA interactions, the following biotin-labeled probes were designed and synthesized by Suzhou Jinweizhi Biotechnology Co., Ltd. (China): (i) a 30-bp wild-type DNA fragment (GTTATAACAGACATACATGCGCTAATATCG) derived from the *SlERF.D2* promoter containing the SlEILs recognition motif (EBS: TACAT), and its mutant version in which the EBS motif was replaced by AAAAA; (ii) a 32-bp wild-type DNA fragment (CGCAAGTAGGCCGCCTCTCCCCCATCTTCTTA) derived from the *SlPP2C1* promoter encompassing the ERF binding site (GCC box: GCCGCC), and its mutant counterpart in which the GCC box was replaced by AAAAAA. All probes were labeled with biotin at the 5′ end. The binding specificity was evaluated using unlabeled wild-type probe fragments (cold competition), biotin-labeled mutant probes (non-specific competition), and MBP protein alone (negative control). Protein purification, binding reaction, and experimental methods were performed as described in a previous study [68]. Primers used for cloning and probe design are provided in Table S1.

### Accession numbers

Gene sequence information was obtained from the Solanaceae Genomics Network (https://solgenomics.sgn.cornell.edu/) and NCBI. *SlActin7* (*Solyc03g078400*/ *NM_001308447.1*), *SlERF.D1* (*Solyc04g051360*/ *XM_004237435.5*), *SlERF.D2* (*Solyc12g056590*/ *NM_001346061.2*), *SlERF.D5* (*Solyc04g012050*/ *XM_004237096.5*), *SlERF.D6* (*Solyc04g071770*/ *XM_004237769.5*), *SlERF.D9* (*Solyc06g068830*/ *XM_004242144.5*), *AtERF110* (*NM_001344871.1*), *AtERF114* (*NM_125582.3*), *SlSGR1* (*Solyc08g080090*), *SlSAG12* (*Solyc02g076910*), *SlEIL1* (*Solyc06g073720*), *SlEIL2* (*Solyc01g009170*), *SlEIL3* (*Solyc01g096810*), *SlEIL4* (*Solyc06g073730*), *SlPP2C1* (*Solyc03g096670*), *SlAREB1* (*Solyc04g078840*), *SlDREB2A* (*Solyc05g052410*).

## Supplementary Material

Web_Material_uhaf267
